# An Open Comparative Study on the Effectiveness of Customized and Non-Customized Sound Therapy for Tinnitus Patients

**DOI:** 10.3390/jcm15124431

**Published:** 2026-06-08

**Authors:** Hongbo Xie, Yuehong Liu, Siyi Yang, Tianyi Ni, Jian Ruan, Yun Jiang, Ziyuan Lin, Lingling Zhou, Songpo He, Zhao Han

**Affiliations:** 1 Department of Otolaryngology-Head and Neck Surgery, Huadong Hospital, Fudan University, Shanghai 200040, China; hbxie22@m.fudan.edu.cn (H.X.);; 2 Geriatric Tinnitus and Deafness Center, Huadong Hospital, Shanghai 200040, China; 3 Department of Otolaryngology, Xuchang Central Hospital, Xuchang 461002, China

**Keywords:** customized sound therapy, non-customized sound therapy, sound therapy effect, subjective tinnitus

## Abstract

**Objectives:** Sound therapy is a common treatment for tinnitus. This study aims to explore the efficacy of non-customized and customized sound therapy for subjective tinnitus. Treatment progress was assessed by comparing changes in monthly scales. The main scales used are the Tinnitus Handicap Inventory, Hospital Anxiety and Depression Scale, and Loudness Visual Analogue Scale. **Methods:** The subjects of this study were patients with tinnitus who visited outpatient from 2024 to 2025. Total of 732 patients were included (customized group, *n* = 653; non-customized group, *n* = 79). Customized sound therapy is tailored to patient’s specific tinnitus condition, while non-customized sound consists of soothing natural sounds. The daily treatment duration was 2 h. Treatment assignment was not randomized; patients were free to choose between customized and non-customized sound therapy, which may have introduced a preference bias. **Results:** The study found that, in both groups of patients, regardless of whether they were treated or not, the severity of tinnitus was positively correlated with the level of anxiety (*p* < 0.01)/depression (*p* < 0.01) and the loudness of tinnitus (*p* < 0.01). Patients with severe tinnitus were more likely to choose customized sound therapy (*p* < 0.01). THI score for the non-customized group showed a downward trend at 1 and 3 months after treatment (*p* < 0.01 at 1 and 3 months). HADS-A score also decreased 1 and 3 months after treatment compared to before treatment (*p* < 0.05 at 1 and 3 months). THI scores for the customized group showed a downward trend at 1, 3, 6, 9, and 12 months after treatment (*p* < 0.001 at 1 and 3 & 6 and 9 & 12 months). HADS-A/D scores decreased at 1, 3, 6, 9 and 12 months after treatment (*p* < 0.01 at 1 & 3 & 6 and 9 & 12 months). The efficacy of the customized group was greater than that of the non-customized group 1 month after treatment (χ^2^ = 6.39, *p* = 0.011). **Conclusions:** Both sound therapies can alleviate tinnitus severity and reduce anxiety in the short term. Customized sound therapy is superior to non-customized therapy in terms of short-term efficacy. Additionally, customized sound therapy is more effective than non-customized therapy in alleviating tinnitus severity and managing anxiety and depression over a longer period. Therefore, customized sound therapy may be a more effective treatment option for subjective tinnitus. However, it should be emphasized that this was an open study in which patients freely chose between the two therapies, which may have introduced bias. Therefore, these findings are not as well founded as those from a randomized controlled trial, and future RCTs are warranted to verify the observed benefits.

## 1. Introduction

Tinnitus is a common symptom in otolaryngology and audiology clinics [1,2]. Epidemiological studies show that tinnitus affects approximately 10–15% of adults [3] and significantly impacts their quality of life [4]. If tinnitus is not intervened and treated in time, it may cause emotional disorders in patients, the two most common of which are anxiety and depression [5,6]. It can be subjective or objective, and its etiology involves various otological and systemic factors, including hearing loss, noise exposure, and certain systemic chronic diseases [7].

Treatment guidelines [8,9] recommend cognitive behavioral therapy (CBT) as an effective treatment for tinnitus [8]. Sound therapy, which shares similar treatment goals with CBT, is commonly used in clinical practice for tinnitus [10]. Sound therapy encompasses several modalities, such as broadband noise masking, notched music, and customized sounds that are tailored to individual tinnitus characteristics [11]. Previous studies on sound therapy have reported mixed results, with some showing significant reductions in tinnitus severity [12] and others demonstrating only modest benefits [13]; sample sizes have varied from small pilot cohorts to larger clinical series, and limitations include heterogeneous protocols and potential placebo effects [14]. Whether customized sounds offer clinically meaningful advantages over non-customized sounds remains unclear, as studies that directly compare these two approaches are scarce.

Previous studies have demonstrated the effectiveness of sound therapy in reducing the neural excitability associated with tinnitus [15]. Recent research suggested personalized treatment of subjective tinnitus using customized sounds [16]. The rationale is that customized sounds can decrease tinnitus-related cortical activity and subjective tinnitus perception [16]. Based on these arguments, this study aimed to compare the therapeutic effects of customized sounds and non-customized sounds on subjective tinnitus. We hypothesized that customized sounds would yield greater improvements in tinnitus severity. The study evaluated the treatment effects from both short-term and long-term perspectives.

## 2. Materials and Methods

### 2.1. Participants

This study included patients who visited the Department of Otolaryngology and Audiology Clinic at Huadong Hospital Affiliated to Fudan University from January 2024 to December 2025, which included the initial preparation phase, the formal data collection phase, and the final data processing and analysis phase: ① 1 January 2024–15 August 2024: This phase was dedicated exclusively to study design, literature review, personnel training, and preliminary experiments (including the selection and refinement of assessment scales); ② 15 August 2024–15 August 2025: This phase involved formal patient data collection and protocol execution.; ③ 15 August 2025–31 December 2025: This final phase was devoted solely to data analysis, interpretation of results, and manuscript preparation. All activities involving human participants commenced only after ethics approval had been obtained and were completed entirely within the approved validity period. The study was approved by the Ethics Committee of Huadong Hospital affiliated to Fudan University on 14 August 2024 (protocol number: 2024K246).

All patients underwent a standardized clinical assessment before enrollment, including a general otorhinolaryngological examination (otoscopy, head and neck examination), pure-tone audiometry (125–8000 Hz), and tympanometry. Brain magnetic resonance imaging (MRI) was performed when clinically indicated to exclude retrocochlear pathology such as acoustic neuroma. The inclusion criteria were: ① age ≥ 14 years; ② main complaint of unilateral or bilateral subjective tinnitus lasting ≥ 6 months; ③ with or without sensorineural hearing loss and normal middle ear function. Exclusion criteria included: ① ear diseases, such as otitis media and acoustic neuroma; ② objective tinnitus, such as middle ear muscle clonus and vascular pulsation tinnitus; ③ systemic diseases that may cause tinnitus symptoms, including hyperthyroidism, hypertension, and diabetes; ④ mental illness or communication disorders; ⑤ patients who were currently receiving or had previously received any form of sound therapy or masking therapy. All patients voluntarily signed the informed consent form to participate in the study. A total of 732 patients were enrolled, with a mean age of 39.82 years and 363 males.

### 2.2. Evaluation of Tinnitus

Tinnitus severity and treatment outcomes were evaluated using the following three scales:

① Tinnitus Handicap Inventory (THI): This 25-item questionnaire evaluates how tinnitus affects daily life across three subscales: functional, emotional, and catastrophic. Each item is scored as “yes” (4 points), “sometimes” (2 points), or “no” (0 points) [17]. The THI score ranges from 0 to 100 points, with a higher score indicating a greater negative impact of tinnitus [18]. Based on the score, patients’ tinnitus can be classified as slight (0–16 points), mild (18–36 points), moderate (38–56 points), severe (58–76 points), or catastrophic (78–100 points) [19]. The Chinese version of the THI has been validated and is widely used [20].
② Hospital Anxiety and Depression Scale (HADS): This screening scale assesses anxiety and depression in patients in general hospitals [21]. It consists of HADS-A for anxiety and HADS-D for depression. Each subscale has 7 questions, scored from 0 to 3 based on the frequency of symptoms in the past month. The subscale score ranges from 0 to 21 points, with a higher score indicating more severe anxiety or depression symptoms [22]. Based on the score, patients’ anxiety or depression levels can be classified as negative (0–7), suspected (8–10), or definite (15–21). The Chinese version of the HADS has been validated [23].
③ Loudness Visual Analogue Scale (VAS): This scale assesses the intensity of tinnitus [24]. The score ranges from 0 to 10, with a higher score indicating a louder tinnitus sound intensity. The VAS is an intuitive, widely used measure.

The changes in THI, HADS-A, HADS-D, and loudness VAS scores were compared before and at 1, 3, 6, 9, and 12 months after treatment in customized sound group and the non-customized sound group.

### 2.3. Patient Education

Prior to the intervention, scientific education and consultation were conducted with patients lasting 15 min. Patients were informed about tinnitus, including its basic information and potential emotional impacts. They were also introduced to various assessment scales and given instructions on how to complete them accurately.

### 2.4. Sound Production and Selection

Our study involves two types of sounds: “customized sounds” and “non-customized sounds.”

Customized sounds are produced by the core software of the “Tinnitus Assistant” app version 4.0.1, which uses artificial intelligence to simulate subjective tinnitus sounds. This software is provided by Sound Ocean Company (Suzhou, Jiangsu Province, China). The relevant customized sound therapy technology is approved by the FDA, and the customized sound therapy method used by this software has been authorized by the China National Intellectual Property Administration. The acquisition of simulated tinnitus sounds occurs through four steps: ① The patient’s tinnitus characteristics are classified into three types: “common tinnitus sounds,” “electronic sounds,” and “daily life sounds.” ② Further categorization is done for each type mentioned in step ①. “Common tinnitus sounds” include tones/ringing, hissing/buzzing, or insect sounds. “Electronic sounds” include various types of electronic/mechanical sounds. “Daily life sounds” include non-human sounds from TVs and the sound of boiling water. ③ Based on the tinnitus type selected in the first two steps, the patient’s tinnitus type and frequency are matched. ④ The patient’s tinnitus loudness is matched to the different volume levels. The obtained simulated tinnitus sound is then input into the tinnitus assistant application, and the computer records the active area of the brain’s reaction to simulate the response to the tinnitus sound. The frequency, loudness, and phase of the sound within the patient’s hearing loss frequency range are edited and adjusted through the inverse phase editing program. This ensures that the frequency and loudness of the customized sound are the same as the simulated tinnitus sound, but the phase is opposite. By utilizing wave superposition characteristics, the superposition of customized sound and tinnitus sound can neutralize and eliminate each other. As the patient’s tinnitus symptoms gradually improve, the customized sound can also stimulate the patient’s brain in reverse, reducing sensitivity to the tinnitus sound [25,26].

The principle of selecting non-customized sounds is to use nature sounds that do not elicit positive or negative emotions in patients. These sounds should be meaningless and gentle. The selection process involves two steps: ① The doctor/audiologist assists the patient in choosing a sound, such as the sound of rain or wind blowing through leaves. ② The doctor/audiologist adjusts the volume to match the loudness of the patient’s tinnitus [27].

### 2.5. Sound Therapy

Patients are advised to use specific headphones (model: EdifierW800BT) for sound therapy in a quiet setting, free from external noise. The recommended duration for sound therapy are 2 h per day. If the loudness or type of tinnitus changes, patients can contact their doctor or audiologist via the app for guidance.

### 2.6. Follow-Up System

The “Tinnitus Assistant” app will follow up with patients using customized groups for up to 12 months. The app will automatically track treatment time [12]. Patients in the non-customized group received treatment and followed up on their own. All patients had a follow-up and consultation system under professional guidance. Before and after treatment, patients completed monthly evaluations, and were required to submit all questionnaires. Throughout the treatment period, patients and doctors/audiologists communicated about treatment-related issues.

### 2.7. Statistical Analysis

All statistical analyses were performed using SPSS version 25.0 (IBM Corp., Armonk, NY, USA). The normality of continuous data was assessed using the Shapiro–Wilk test. For normally distributed variables, between-group comparisons at each time point were performed using independent-samples *t*-tests, and within-group changes over time were analyzed using repeated-measures analysis of variance (ANOVA). For non-normally distributed data, the Mann–Whitney U test and the Friedman test were used as appropriate. Categorical variables were compared using the chi-square test or Fisher’s exact test. A two-tailed *p*-value < 0.05 was considered statistically significant.

## 3. Results

### 3.1. Participants Characteristics

In our study, 732 patients met the criteria and were included across both treatment groups. There were no statistically significant differences between the two treatment groups regarding the distribution of sex, age, or tinnitus duration (*p* > 0.05). Tinnitus was the main symptom experienced by these patients. Pre-treatment characteristics are shown in Table 1.

The pre-treatment THI scale scores divided the patients into 5 levels of tinnitus severity. A comparison of tinnitus severity scores (THI scores) between the two groups showed a statistically significant difference (*p* = 0.014), indicating that patients with severe tinnitus were more likely to choose customized sound therapy.

Pre-therapy, the proportion of patients with clinically significant anxiety (HADS-A ≥ 8) was significantly higher in the customized group than in the non-customized group (65.70% vs. 58.23%, *p* = 0.043). The proportion of patients with depression (HADS-D ≥ 8) did not differ significantly between the two groups (41.04% vs. 31.65%, *p* = 0.459). The composite indicator of emotional disorder (HADS-A ≥ 8 or HADS-D ≥ 8) likewise did not differ significantly between groups (71.98% vs. 62.03%, χ^2^ = 3.38, *p* = 0.066). This may suggest that tinnitus patients with anxiety disorders are more likely to choose customized sound therapy; the severity of tinnitus is positively correlated with emotional disorders, especially anxiety.

### 3.2. Tinnitus Severity Is Associated with Levels of Anxiety/Depression and Loudness VAS Scores

As shown in Table 2, pre-therapy, THI scores showed strong positive correlations with HADS-A, HADS-D, and VAS in both groups, with Spearman’s rho values ranging from 0.56 to 0.65 (*p* < 0.01). At 3 months post-treatment, these correlations remained strong. In the non-customized group, the correlation between THI and HADS-A reached 0.77, while in the customized group it was 0.63 (*p* < 0.01). The associations between VAS loudness and the emotional scales were consistently low to moderate. Pre-therapy, VAS correlated with HADS-A at 0.28 in the non-customized group and 0.39 in the customized group; with HADS-D, the corresponding rho values were 0.40 and 0.36. At 3 months, VAS and HADS-D showed a non-significant correlation of 0.29 in the non-customized group. These findings indicate that tinnitus severity is robustly linked to emotional distress, whereas the relationship between perceived loudness and mood symptoms is substantially weaker.

### 3.3. Curative Effect of Customized and Non-Customized Sound Therapy in Treating Subjective Tinnitus

Our evaluation of the efficacy of the two treatment groups is based on changes in THI levels before and after treatment. As shown in Table 3, the median THI of patients in the non-customized group decreased from 44 (26, 72) to 34 (16, 53), while the median THI of patients in the customized group decreased from 54 (36, 76) to 38 (24, 50).

In the non-customized group, THI scores at 1 month and 3 months were significantly lower than those before treatment (*p* = 0.002 at 1 month; *p* < 0.001 at 3 months). However, THI scores at 3 months did not differ significantly from those at 1 month (*p* = 0.099).

In the customized group, THI scores at 1, 3, 6, 9, and 12 months after treatment were significantly lower than those before treatment (all *p* < 0.001). Unlike the non-customized group, THI scores in the customized group at 3, 6, and 9 months were significantly lower than those at 1 month (*p* < 0.001 at 3 and 6 months; *p* = 0.004 at 9 months).

The results showed that the THI scores of both groups of patients decreased after treatment. This suggests that, after a period of regular sound therapy—whether customized or non-customized—the severity of tinnitus experienced by patients can be alleviated to a certain extent. Furthermore, we found that patients in the customized sound therapy group showed a further decrease in their THI scores after a longer treatment period, while the non-customized sound therapy group did not show any further decrease in their THI scores. This may suggest that customized sound therapy may provide a more enduring therapeutic effect for patients suffering from tinnitus.

**Table 3:** THI treatment outcomes after 12 months of non-customized sound therapy and customized sound therapy.

Treatment Time	Groups	THI Median (Q1, Q3)	*p*-Value (vs. BL) ^a^	*p*-Value (vs. 1 M) ^b^
BL	Non-Customized	44 (26, 72)	/	/
	Customized	54 (36, 76)	/	/
1 M	Non-Customized	36 (24, 58)	0.002	/
	Customized	42 (26, 58)	<0.001	/
3 M	Non-Customized	34 (16, 53)	<0.001	0.099
	Customized	36 (22, 53)	<0.001	<0.001
6 M	Non-Customized	32 (10, 42)	/	/
	Customized	38 (24, 50)	<0.001	<0.001
9 M	Non-Customized	/	/	/
	Customized	40 (24, 52)	<0.001	0.004
12 M	Non-Customized	/	/	/
	Customized	42 (16, 50)	<0.001	0.059

**Figure 1:**
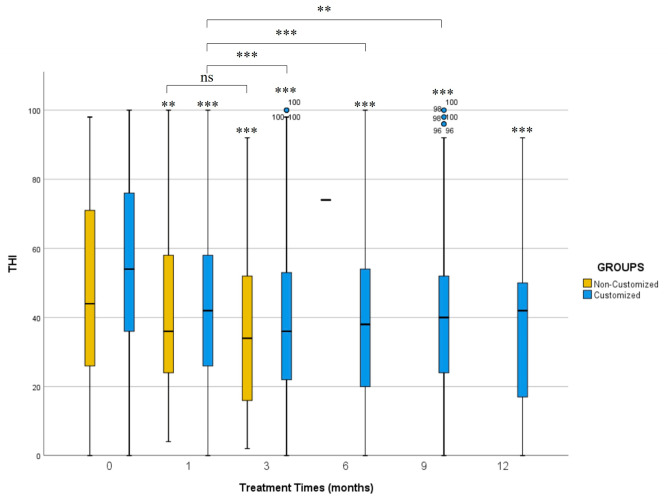
Box plots of Tinnitus Handicap Inventory (THI) total scores at baseline and during follow-up in the customized and non-customized sound therapy groups. note: *p*-values are from comparisons using the Wilcoxon signed-rank test. ns. *p* > 0.05, ** *p* < 0.01, *** *p* < 0.001 (two-tailed). THI = Tinnitus Handicap Inventory.

Values are median (Q1, Q3). P-values are from comparisons using the Wilcoxon signed-rank test. The box plots for THI scores are shown in Figure 1. ^a^: Compared with Baseline. ^b^: Compared with 1 Month. BL = Baseline; M = Month; THI = Tinnitus Handicap Inventory.

### 3.4. Efficacy of Customized and Non-Customized Sound Therapy for Anxiety and Depression in Patients with Tinnitus

#### 3.4.1. HADS-A (Anxiety)

In the non-customized group, the median HADS-A score decreased from 8 (6, 11) to 6.5 (2.75, 8). There was a statistically significant difference in HADS-A scores before treatment and at 1 and 3 months after treatment (*p* = 0.026 at 1 month; *p* = 0.005 at 3 months), but no significant difference at 6 months after treatment (*p* > 0.05). In addition, there was no statistically significant difference in HADS-A scores at 3 months post-treatment compared to 1 month after treatment (*p* = 0.780).

In the customized group, the median HADS-A score decreased from 9 (7, 12) to 7 (5, 9). There was a statistically significant difference in HADS-A scores before treatment and at 1, 3, 6, 9, and 12 months after treatment (all *p* < 0.001). Additionally, HADS-A scores at 6 and 9 months after treatment were significantly lower than those at 1 month after treatment (*p* = 0.017 at 6 months; *p* = 0.028 at 9 months).

**Figure 2:**
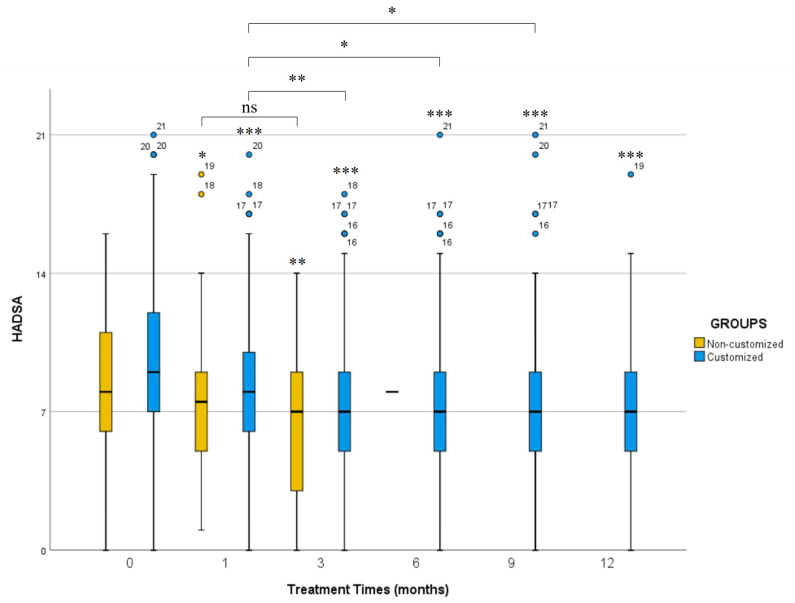
Box plots of Hospital Anxiety and Depression Scale-Anxiety (HADS-A) scores at baseline and during follow-up in the customized and non-customized sound therapy groups. note: *p*-values are from comparisons using the Wilcoxon signed-rank test. ns. *p* > 0.05, * *p* < 0.05, ** *p* < 0.01, *** *p* < 0.001 (two-tailed). HADS = Hospital Anxiety and Depression Scale; A = anxiety subscale.

As shown in Table 4 and Figure 2, the HADS-A scores of both groups of patients decreased after treatment. Furthermore, similar to the findings above, patients in the customized sound therapy group showed a further decline in their HADS-A scores after receiving longer-term treatment; in contrast, patients in the non-customized sound therapy group did not show any further decline in their HADS-A scores.

#### 3.4.2. HADS-D (Depression)

In the non-customized group, HADS-D scores at 1 and 3 months did not differ significantly from those before treatment (*p* = 0.162 at 1 month; *p* = 0.056 at 3 months).

In the customized group, the median HADS-D score decreased from 6 (3, 10) to 4 (2, 8). HADS-D scores at 1, 3, 6, 9, and 12 months were significantly lower than those before treatment (*p* < 0.001 at 1, 3, 6, and 9 months; *p* = 0.002 at 12 months). Furthermore, HADS-D scores at 3 and 6 months were significantly lower than those at 1 month (*p* = 0.009 at 3 months; *p* = 0.001 at 6 months).

**Figure 3:**
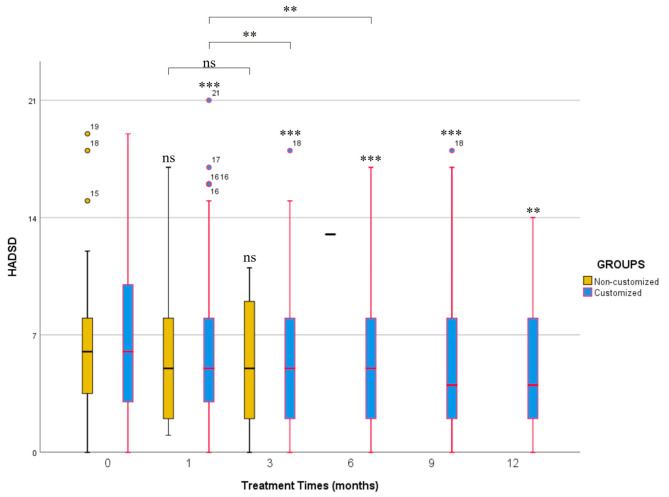
Box plots of Hospital Anxiety and Depression Scale-Depression (HADS-D) scores at baseline and during follow-up in the customized and non-customized sound therapy groups. note: *p*-values are from comparisons using the Wilcoxon signed-rank test. ns. *p* > 0.05, ** *p* < 0.01, *** *p* < 0.001 (two-tailed). HADS = Hospital Anxiety and Depression Scale; D = depression subscale.

As shown in Table 5 and Figure 3, patients in the non-customized sound therapy group did not experience a significant decrease in their HADS-D scores after treatment, while patients in the customized sound therapy group experienced a significant decrease in their HADS-D scores. Furthermore, patients in the customized sound therapy group showed a further downward trend in their HADS-D scores after receiving longer-term treatment.

Based on a comprehensive analysis of the foregoing, these findings suggest that regular application of non-customized sound therapy may alleviate anxiety in tinnitus patients, at least in the short term, but does not appear to mitigate depression. In contrast, regular application of customized sound therapy appears capable of alleviating both anxiety and depression. Furthermore, for tinnitus patients concurrently affected by emotional disorders, customized sound therapy may offer more enduring therapeutic benefits.

### 3.5. Customized Sound Therapy Is More Effective in Treating Subjective Tinnitus than Non-Customized

After 1 month of treatment, we observed that 45.1% of patients in the customized group experienced a therapeutic effect on the severity of tinnitus, significantly higher than the 29.7% in the non-customized group. Statistical analysis confirmed this finding (see Table 6 for details).

## 4. Discussion

This study compared the therapeutic effects of customized and non-customized sound therapy on subjective tinnitus. The main findings were as follows. First, both sound therapy modalities reduced tinnitus severity, but customized sound therapy produced a further progressive decline with longer treatment, whereas non-customized sound therapy did not. Second, customized sound therapy was associated with a higher response rate at 1 month. Third, customized sound therapy significantly alleviated both anxiety and depression, and these improvements showed a further progressive reduction with longer treatment; in contrast, non-customized sound therapy only partially relieved anxiety in the short term and had no significant effect on depression, and no progressive improvement in emotional symptoms was observed with extended treatment. Finally, tinnitus loudness showed only weak-to-moderate correlations with emotional distress, consistent with previous reports indicating that perceived loudness is not the primary driver of tinnitus-related emotional disturbance [28].

Tinnitus refers to the perception of sound without an external physical sound source [29] and can have a significant impact on patients’ daily lives, often leading to insomnia and emotional disorders, especially in severe cases. As a result, affected individuals may exhibit irrational behaviors such as self-harm or even suicide [30]. Doctors and audiologists specializing in tinnitus need to address this problem.

The severity of tinnitus was evaluated using the THI [18] and emotional symptoms were assessed with HADS A/D [21]. The Chinese versions of these instruments have been validated and widely used in clinical settings [31]. Tinnitus loudness was rated on a VAS, a well-established method for assessing subjective tinnitus intensity [32].

Due to the complex and incompletely understood mechanism of tinnitus, no curative treatment currently exists. CBT is recommended by clinical guidelines as an effective intervention for tinnitus [33], helping patients learn to live with tinnitus and reduce its impact [8,9]. The principles and objectives of sound therapy are similar to those of CBT, but sound therapy is easier to implement [34]. In this study, two types of sound therapy were compared. Non-customized sounds are emotionally neutral nature sounds selected by the clinician. Customized sounds are individually tailored using artificial intelligence-based software that simulates the patient’s tinnitus percept and generates an inverse-phase sound designed to reduce tinnitus-related neural excitability [25,26]. One hypothesized mechanism of tinnitus involves abnormal excitation of the cerebral cortex and central remodeling following damage to the peripheral auditory system [35]. It is possible that the customized sound stimulation may help remodel relevant neurons in the auditory cortex over time [36]. Additionally, based on our clinical observations, patients with higher tinnitus severity tended to receive customized sound therapy. This may be because more severe tinnitus is often accompanied by greater emotional distress, leading these patients to seek treatment that directly targets the tinnitus percept itself. The customized approach, by generating a sound that can neutralize the tinnitus percept, may provide a more immediate subjective experience of relief during the initial stages of treatment, which could enhance patients’ motivation and adherence.

According to our findings, both sound therapy methods can temporarily reduce the severity of tinnitus in patients. Non-customized sound therapy results in a decrease in severity of tinnitus after short-term treatment, but there is no additional reduction in severity after longer treatment. On the other hand, patients receiving customized sound therapy experience a decrease in severity of tinnitus after short-term treatment, and this reduction further improves after longer treatment. Additionally, we compared the effectiveness of both treatment groups 1 month after treatment. It was discovered that customized sound therapy was more effective than non-customized sound therapy. This may be due to the fact that non-customized sound therapy can divert attention away from tinnitus, thus reducing its severity to some extent. Over time, patients may find that these nature sounds alone are insufficient to continuously alleviate the impact of tinnitus. In contrast, customized sound therapy can diminish the impact of tinnitus on patients in both the short and long term.

Research has shown that tinnitus is frequently associated with emotional disorders, particularly anxiety and depression, which may reflect overlapping brain networks involving the limbic and auditory systems [37]. In the present study, the severity of tinnitus was positively correlated with both anxiety and depression, with anxiety being more prevalent than depression. These patterns are broadly consistent with the existing literature [38]. In terms of treatment effects, non-customized sound therapy partially alleviated anxiety in the short term but did not significantly reduce depression. In contrast, customized sound therapy significantly reduced both anxiety and depression, and these improvements were maintained for 6 months or longer. This difference may be attributable to the greater and more sustained reduction in tinnitus severity achieved with customized sounds: when patients perceive meaningful and lasting tinnitus relief, the associated emotional distress may also subside. Conversely, when treatment fails to produce lasting tinnitus improvement, anxiety and depression may re-emerge. Notably, we also found that tinnitus loudness showed only weak-to-moderate correlations with emotional symptoms, suggesting that emotional distress in tinnitus patients is driven more by tinnitus-related handicap than by perceived loudness per se, a finding in agreement with previous reports [28].

Our research is based on a comprehensive follow-up system. Patients in the customized group are provided with the “Tinnitus Assistant” app to monitor their daily treatment and monthly follow-up. For non-customized group patients, they should be more diligent in completing their daily treatment plans. The follow-up system is available to all patients, who can complete the scale themselves and upload relevant data. If they need consultation, they can leave messages in the follow-up system, and the doctor/audiologist will contact them for consultation. In previous studies, telephone follow-up was the common method [34,39]. However, for larger sample clinical studies, this method is almost impossible to implement. When combined with an effective follow-up system, the cure rate can be significantly improved. Therefore, our research provides a crucial follow-up system for patients.

This study has several limitations. First, it was not a randomized controlled trial; treatment allocation was based on clinical choice, which may have introduced selection bias. This limitation is reflected in the significant baseline imbalances between groups, including higher tinnitus severity and greater anxiety prevalence in the customized group. Although causality cannot be established, it may be cautiously inferred that patients with more severe tinnitus and more pronounced emotional disturbance were more inclined to choose customized sound therapy, which in turn contributed to the observed baseline differences. Second, the sample size of the non-customized group was considerably smaller than that of the customized group, which may reduce statistical power and affect the stability of between-group comparisons. Third, loss to follow-up in the non-customized group approached 100% by the 6-month time point, which not only limited long-term comparisons between the two modalities but may also partly reflect reduced treatment satisfaction or waning confidence in the non-customized approach. Although this observation should be interpreted with caution, it is consistent with the result that non-customized sound therapy did not produce sustained improvement. Finally, the study was conducted at a single center, which may limit the generalizability of the findings. Minimizing attrition, extending follow-up, and conducting randomized controlled trials in future studies would help yield more robust conclusions.

## 5. Conclusions

Our study compares two methods of sound therapy: non-customized and customized. The results indicate that both methods can effectively reduce the severity of tinnitus and alleviate anxiety in patients in the short term. However, customized sound therapy demonstrates superior efficacy compared to non-customized sound therapy in the short term. Furthermore, it proves to be more effective in alleviating the severity of tinnitus, anxiety, and depression disorders over a longer duration. However, it should be emphasized that this was an open study in which patients freely chose between the two therapies, which may have introduced bias. Therefore, these findings are not as well founded as those from a randomized controlled trial, and future RCTs are warranted to verify the observed benefits.

## Figures and Tables

**Table 1:** Pre-therapy demographic and clinical characteristics of two groups of patients.

Groups	Non-Customized (N = 79)	Customized (N = 653)	*p*-Value
Characteristics			
Sex, male, n (%)	43 (54.43)	320 (49.00)	0.362
Age (years), mean ± SD	39.94 ± 12.05	39.80 ± 11.30	0.996
Tinnitus duration (years) (mean ± SD)	3.09 ± 3.57	3.16 ± 4.67	0.597
Tinnitus severity, n (%)			0.014
Slight (0–16)	11 (13.92%)	30 (4.59%)	
Mild (18–36)	18 (22.78%)	138 (21.13%)	
Moderate (38–56)	25 (31.65%)	191 (29.25%)	
Severe (58–76)	12 (15.19%)	148 (22.66%)	
Catastrophic (78–100)	13 (16.46%)	146 (22.36%)	
Anxiety proportion (HADS-A ≥ 8), n (%)	46 (58.23)	429 (65.70)	0.043
Depression proportion (HADS-D ≥ 8), n (%)	25 (31.65)	268 (41.04)	0.459
Emotional disorder proportion (HADS-A or D ≥ 8), n (%)	49 (62.03)	470 (71.98)	0.066 ^a^

Values are presented as mean ± SD or n (%). Between-group comparisons: continuous variables (age, tinnitus duration, tinnitus severity, anxiety and depression proportion) were assessed for normality using the Shapiro–Wilk test; *p*-values were derived from Mann–Whitney U tests for non-normally distributed variables. Categorical variables (sex, emotional disorder) were compared using Pearson’s Chi-squared test. ^a^ Overall comparison of emotional disorder proportion: χ^2^ = 3.38, *p* = 0.006 (Pearson’s Chi-squared test). HADS: Hospital Anxiety and Depression Scale; A: anxiety subscale; D: depression subscale.

**Table 2:** Correlation between the severity of tinnitus and levels of depression/anxiety/VAS in the two groups.

Groups	Non-Customized				Customized			
Pre-Therapy	THI	HADS-A	HADS-D	VAS	THI	HADS-A	HADS-D	VAS
THI	1	0.585 **	0.590 **	0.561 **	1	0.652 **	0.566 **	0.609 **
HADS-A	-	1	0.673 **	0.279 *	-	1	0.537 **	0.390 **
HADS-D	-	-	1	0.396 **	-	-	1	0.356 **
VAS	-	-	-	1	-	-	-	1
3M-post-therapy	THI	HADS-A	HADS-D	VAS	THI	HADS-A	HADS-D	VAS
THI	1	0.766 **	0.613 **	0.608 **	1	0.630 **	0.532 **	0.605 **
HADS-A	-	1	0.668 **	0.449 **	-	1	0.570 **	0.411 **
HADS-D	-	-	1	0.293	-	-	1	0.379 **
VAS	-	-	-	1	-	-	-	1

THI = Tinnitus Handicap Inventory; HADS = Hospital Anxiety and Depression Scale; A = anxiety subscale; D = depression subscale; VAS = visual analogue scale for tinnitus loudness. Values are Spearman’s rho. * *p* < 0.05, ** *p* < 0.01 (two-tailed).

**Table 4:** HADS-A treatment outcomes after 12 months of non-customized sound therapy and customized sound therapy.

Treatment Time	Groups	HADS-A Median (Q1, Q3)	*p*-Value (vs. BL)	*p*-Value (vs. 1 M) ^a^
BL	Non-Customized	8 (6, 11)	/	/
	Customized	9 (7, 12)	/	/
1 M	Non-Customized	7.5 (5, 9)	0.026	/
	Customized	8 (6, 10)	<0.001	/
3 M	Non-Customized	7 (2.5, 9.5)	0.005	0.780
	Customized	7 (5, 9)	<0.001	0.002
6 M	Non-Customized	6.5 (2.75, 8)	/	/
	Customized	7 (5, 9)	<0.001	0.017
9 M	Non-Customized	/	/	/
	Customized	7 (5, 9)	<0.001	0.028
12 M	Non-Customized	/	/	/
	Customized	7 (5, 9)	<0.001	0.059

Values are median (Q1, Q3). *p*-values are from comparisons using the Wilcoxon signed-rank test. The box plots for HADS-A scores are shown in Figure 2. ^a^: Compared with 1 Month. BL = Baseline; M = Month; HADS = Hospital Anxiety and Depression Scale; A = anxiety subscale.

**Table 5:** HADS-D treatment outcomes after 12 months of non-customized sound therapy and customized sound therapy.

Treatment Time	Groups	HADS-D Median (Q1, Q3)	*p*-Value (vs. BL)	*p*-Value (vs. 1 M) ^a^
BL	Non-Customized	6 (3, 8)	/	/
	Customized	6 (3, 10)	/	/
1 M	Non-Customized	5 (2, 8)	0.162	/
	Customized	5 (3, 8)	<0.001	/
3 M	Non-Customized	5 (2, 9)	0.056	0.222
	Customized	5 (2, 8)	<0.001	0.009
6 M	Non-Customized	5 (1, 8)	/	/
	Customized	5 (2, 8)	<0.001	0.001
9 M	Non-Customized	/	/	/
	Customized	4 (2, 8)	<0.001	0.058
12 M	Non-Customized	/	/	/
	Customized	4 (2, 8)	0.002	0.175

Values are median (Q1, Q3). *p*-values are from comparisons using the Wilcoxon signed-rank test. The box plots for HADS-D scores are shown in Figure 3. ^a^: Compared with 1 Month. BL = Baseline; M = Month; HADS = Hospital Anxiety and Depression Scale; D = depression subscale.

**Table 6:** Treatment response in the two groups at 1 month after treatment.

Therapy	Valid	Invalid	Total	χ^2^	*p*-Value
Non-customized	22 (29.7%)	52 (70.3%)	74	6.390	0.011
Customized	279 (45.1%)	339 (54.9%)	618		
Total	301	391	692		

A patient was considered a valid responder if their THI severity grade decreased by ≥1 level at 1 month compared with baseline. Between-group comparison was performed using Pearson’s Chi-squared test.

## Data Availability

The data presented in this study are available on request from the corresponding author.
